# miRNAminer: A tool for homologous microRNA gene search

**DOI:** 10.1186/1471-2105-9-39

**Published:** 2008-01-23

**Authors:** Shay Artzi, Adam Kiezun, Noam Shomron

**Affiliations:** 1 Massachusetts Institute of Technology, 77 Massachusetts Avenue, Cambridge, MA 02139, USA

## Abstract

**Background:**

MicroRNAs (miRNAs), present in most metazoans, are small non-coding RNAs that control gene expression by negatively regulating translation through binding to the 3'UTR of mRNA transcripts. Previously, experimental and computational methods were used to construct miRNA gene repositories agreeing with careful submission guidelines.

**Results:**

An algorithm we developed – miRNAminer – is used for homologous conserved miRNA gene search in several animal species. Given a search query, candidate homologs from different species are tested for their known miRNA properties, such as secondary structure, energy and alignment and conservation, in order to asses their fidelity. When applying miRNAminer on seven mammalian species we identified several hundreds of high-confidence homologous miRNAs increasing the total collection of  (miRbase) miRNAs, in these species, by more than 50%. miRNAminer uses stringent criteria and exhibits high sensitivity and specificity.

**Conclusion:**

We present – miRNAminer – the first web-server for homologous miRNA gene search in animals. miRNAminer can be used to identify conserved homolog miRNA genes and can also be used prior to depositing miRNAs in public databases. miRNAminer is available at .

## Background

MicroRNAs (miRNAs) are short, ~22 nt non-coding RNAs that control gene expression. miRNAs bind to the 3'UTRs of their regulated mRNA transcripts to facilitate mRNA degradation or translation inhibition [1,2]. miRNAs are present in most metazoans and are thought to regulate a diverse range of biological processes [3,4]. miRNA genes' evolution is unique since they mostly emerge through duplication events [5]; exhibit most often unidirectional evolution [6]; are generally gained and not lost [7]; show several cases of rapid evolution in primates [8,9]; are rarely changed due to functional constraints [10]; and, show relatively rare evolutionary acquisition events (accounted by their relatively small numbers).

miRNA predictions based on computational methods, which followed initial extensive cloning efforts, are based on the secondary structure of the miRNA, its phylogenetic conservation and thermodynamic stability [11,12]. miRNA gene repositories are constantly expanding giving rise to more than 3500 reported miRNAs in more than 30 animal species (Sanger miRbase database, Version 10.0 [13,14]). However, it is apparent that even this comprehensive repository is far from completion, accounted by the very few miRNAs listed for dog (6) and chimpanzee (83), compared to human (533), to name two examples. Since these differences cannot be accounted merely by species-specific miRNAs, we saw the need for a computational tool for miRNA homologous searches.

## Implementation

We present miRNAminer, a tool for automatic identification of homolog miRNAs based on a given user defined query miRNA. The tool exploits numerous characteristics of miRNAs: high conservation of precursor sequences, very high conservation of mature sequences (particularly in the seed region, nt 2–8 [15]), and hairpin secondary structure with high folding energy and base pairing. miRNAminer first uses BLAST [16] to select candidate matches and ranks them according to their e-values. Then it employs a series of rigorous filters to improve specificity.

An input query consists of a precursor miRNA, mature miRNA, a set of filter threshold values and the number of best-fitted results requested in the output. We designed miRNAminer's algorithm to maximize specificity of matches. This is because the designed application of miRNAminer is to identify homolog matches after a miRNA has been experimentally confirmed. We estimated the default values presented below so that each filter by itself selects 95% of known miRNAs in training genomes (criteria was also based on [17]).

**Figure 1:**
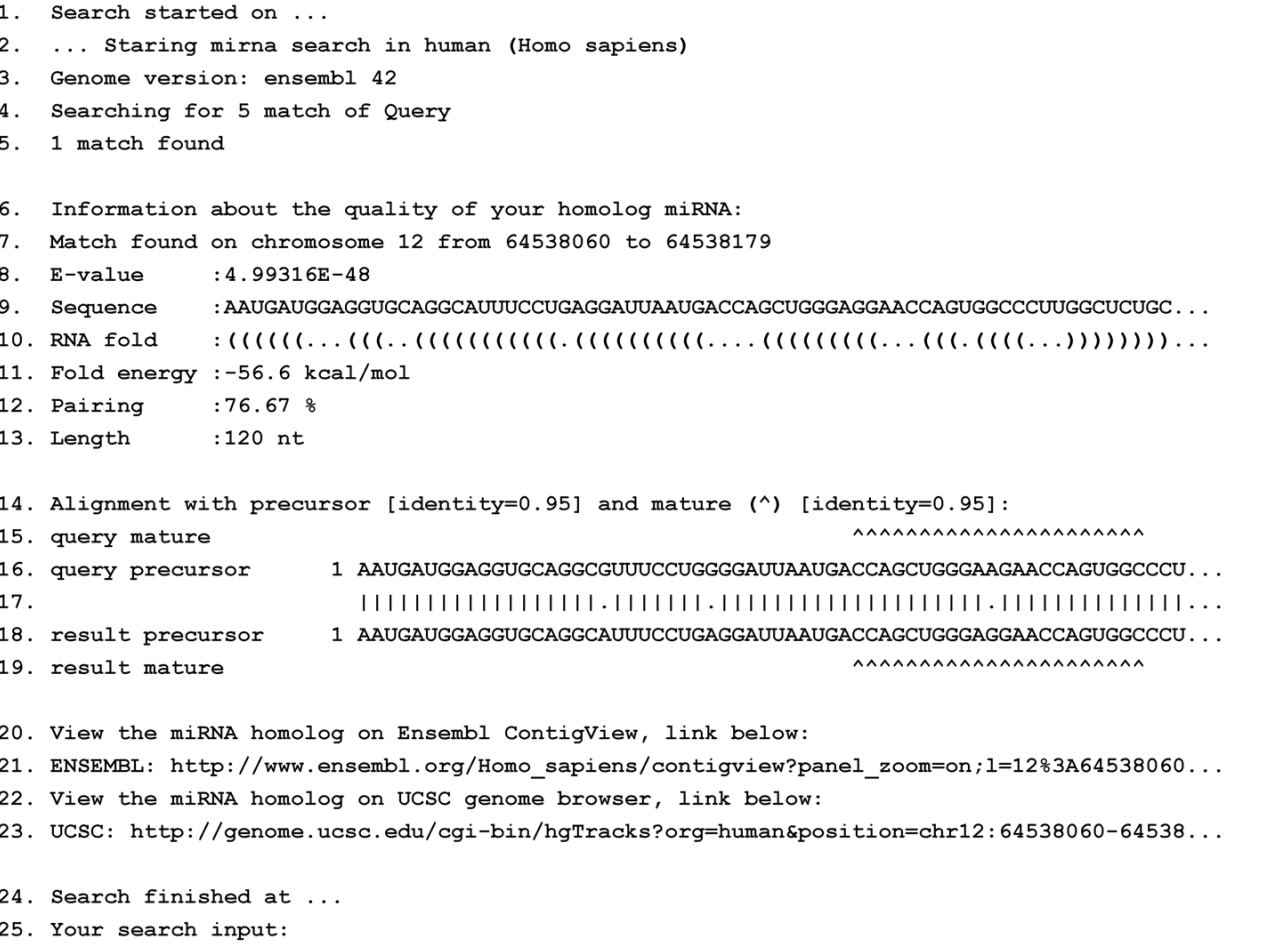
**A sample output of miRNAminer**. Indicated are: search start and end times (rows 1 and 24, respectively) and the species and assembly searched (rows 2–3); whether a match (a miRNA homolog), or matches, that passed the input criteria were found (rows 4–5); information about the quality of the homolog miRNA match such as BLAST e-value, genomic location, sequence, RNA fold and energy, pairing, length and alignment with input sequence (rows 6–19). A hyperlink to the genomic locus of the miRNA homolog is also provided through Ensembl ContigView [18] or UCSC Genome Browser [43] (rows 20–23) and a copy of the users' input data (row 25).

miRNAminer's algorithm follows these steps: (i) Use BLAST [16] to find matches in target genomes (the whole precursor miRNA from the query is used); (ii) Filter with e-value threshold (default 0.05 per chromosome); (iii) Extend the match by adding flanking nucleotides (default 50) up- and down-stream from the match (Ensembl genome database; [18]). Examine all possible extensions of the match within threshold length (default min 70 nt, max 180 nt); (iv) Filter with RNA secondary folding energy threshold (default -25 kcal/mole; RNAfold with options "-p -d2 -noLP" [19]); (v) Filter with minimal base-pairing threshold (default 55% pairing; with 20 gap penalty and 0.5 extension penalty); (vi) Filter with requirement for hairpin-shape secondary structure; (vii) Filter with alignment of precursor sequences (default 56% identity); (viii) Filter with alignment of mature miRNA sequences (default 80% identity); (ix) Filter with maximum number of mismatches in mature miRNA sequences (default 3 nt); (x) Filter with conservation of seed (2–8 nt, required 100% conservation [15]); (xi) Filter with position of mature miRNA on the hairpin (max 4 nt overlap of mature sequence and hairpin loop). miRNAminer's output includes detailed analysis of the identified genomic region(s) that passed the selected threshold criteria (Figure 1). Currently, miRNAminer supports searches in 10 metazoan genomes. We will regularly add additional genomes upon their release. After the query is issued, results are usually available within a minute (though this depends on the number of results requested) and can either be viewed on the screen or requested to be sent by email.

## Results

We used miRNAminer to perform a comprehensive homology search for miRNA precursors in seven species (human, chimpanzee, mouse, rat, dog, cow and opossum). For the search, we used all 2925 vertebrate miRNAs listed in the Sanger miRNA registry (release 9.0 of October 2006). Table 1 shows the summary information of miRNAs listed in the Sanger registry and of new, or non-registered, miRNAs identified by our method. We identified 790 non-miRbase registered miRNAs with major contributions to chimp (*P. troglodytes*), dog (*C. familiaris*) and cow (*B. taurus*), vastly increasing their known miRNA repertoire and possibly opening new research facets in these species (see Additional file 1[20]). Table 2 presents the miRNA candidates that our method identifies in human (*H. sapiens*). It is of interest that 22 new candidate miRNAs in human were identified, despite many previous exhaustive human miRNA identification studies [8,21], possibly due to recent non-human/primate miRNAs identification [22], updated assembly of the human genome (Ensembl *H. sapiens *genome release 42 used here was updated in October 2006), and the modification of search parameters as implemented in miRNAminer.

**Table 1:** Known registered miRbase miRNAs and new candidates identified by miRNAminer.

Genome	miRbase 9.0	Newly identified	Sum
Human	474	22	496
Chimpanzee	83	251	334
Mouse	373	31	404
Rat	234	74	308
Dog	6	228	234
Cow	98	131	229
Opossum	107	53	160

Total	1375	790	2165

The column 'miRNA registry 9.0' shows the number of miRNAs listed for the given species in the Sanger miRNA registry release 9.0. The column 'Newly identified' shows the number of miRNAs candidates identified by miRNAminer.

**Table 2:** Candidate non-registered miRNAs identified by miRNAminer in  human.

Original information		Results in *H. sapiens*				RNA fold		Identity with original	
miRNA	length (nt)	Chr	Position	Length (nt)	e-value	base pair %	ΔG	mature %	precursor %
mmu-mir-759	98	13	52282180	96	3.39E-048	72.9	-32	100	100
mmu-mir-763	120	12	64538060	120	4.99E-048	76.7	-57	95	95
mmu-mir-760	119	1	94084955	120	9.15E-048	66.7	-55	100	95
mmu-mir-708	109	11	78790709	109	1.66E-041	62.4	-50	100	94
*mmu-mir-543*	76	14	100568079	72	7.11E-027	61.1	-22	100	95
*rno-mir-543*	80	14	100568079	72	7.46E-027	61.1	-22	100	96
mmu-mir-670	100	11	43537789	89	7.67E-025	71.9	-36	100	91
mmu-mir-762	76	16	30812726	72	1.45E-024	77.8	-54	91	94
mmu-mir-764	108	X	113780174	96	3.83E-024	66.7	-42	95	91
mmu-mir-675	84	11	1974559	84	5.96E-022	71.4	-53	95	92
mmu-mir-711	82	3	48591339	74	1.24E-014	59.5	-31	91	88
mmu-mir-665	94	14	100411119	86	3.10E-014	65.1	-39	91	87
rno-mir-664	59	1	218440516	70	9.59E-012	60	-26	95	92
*mmu-mir-322*	95	X	133508324	91	2.72E-009	76.9	-47	95	85
*rno-mir-322*	95	X	133508327	87	2.72E-009	80.5	-45	95	85
mmu-mir-718	88	X	152938565	70	9.78E-009	71.4	-39	90	87
mmu-mir-709	88	3	186851919	71	1.21E-005	62	-27	100	70
mmu-mir-466	73	2	35362302	78	4.60E-005	61.5	-21	95	72
rno-mir-292	82	19	58982746	70	8.80E-004	65.7	-31	86	77
mmu-mir-669	97	17	69204319	71	1.33E-003	62	-22	90	86
mmu-mir-705	82	22	45887964	72	2.70E-003	58.3	-22	100	58
mmu-mir-207	79	17	73276373	70	4.10E-003	60	-25	96	62
mmu-mir-720	64	3	165541839	71	3.05E-002	59.2	-21	100	89
mmu-mir-761	76	1	110433694	71	4.76E-002	62	-31	81	84

The results are sorted according to e-values (based on BLAST search; [16]). Lengths refer to lengths of miRNA precursors. For each result, the table shows position, RNA fold quality and the identity ratio of the discovered miRNA candidates to the homolog in the original species. The existence of two occurrences for miR-322 and miR-543 (italicized) indicates that two origins (mouse and rat) of the same miRNA were found in *Homo sapiens*.

**Figure 2:**
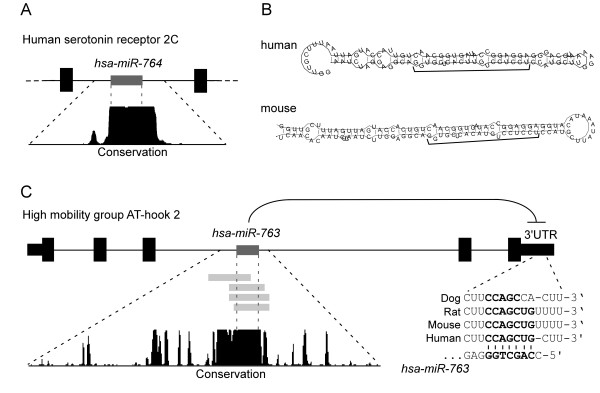
**Two examples of a non-miRbase registered miRNA identified using our miRNAminer web-server**. (A) Human miR-764 was identified using miRbase mouse miR-764 sequence as input (and default parameters) for miRNAminer search. The output reported a homolog (presumably hsa-miR-764), which is located in the second intron of human serotonin receptor 2C (HTR2C; NM 000868). The mouse miRNA homolog is located in an intron of the same gene (HTR2C; NM 008312) suggesting an evolutionary conserved co-expression of miRNA and its host gene [27-29]. High conservation is seen in this region (mountain-like graph derived from UCSC Genome Browser 17 species multiZ alignment; [43]). Black rectangles represent exons (shorter rectangles in C are UTRs), lines are introns and dark-grey rectangles are miRNA genes. (B) RNA secondary structure of both the identified human (top) and mouse (bottom) miR-764 exhibit similar thermodynamic stability (41.8/49.9 kcal/mol, respectively) and structures (mature miRNA region is underlined). Human miR-764 homolog was also identified by Berezikov [21]. (C) Non-registered (miRbase) human miR-763 is highly conserved among vertebrate species and can potentially bind its own host gene. On top; a schematic non-scaled representation of the HMGA2 transcript (NM 003483; human miR-763 is in dark-grey; conservation plot as shown in A). Expressed Sequence Tags (ESTs; light-grey bars) are evidence for the expression of this particular genomic region. ESTs from top to bottom: BM715067 (isolated from eye-related tissue); BJ997562 (isolated from wilms tumor tissue); BU39975 (isolated from eye-related tissue); AI935081 (tissue source unknown). On the right; the potential binding site of miR-763 in HMGA2 3'UTR (nt 2–8 of the miRNA; positions 2192–2198) is conserved to human, mouse and rat.

We compared our predicted miRNAs (in the human species; Table 2) to other prediction methods. We found that 18 and 36% of our miRNAs are contained within RNAmicro [23,5] and Berezikov 2005 [24] databases, respectively, out of almost 3000 and 1000 miRNAs in each set, respectively. The overlap is not extensive however even when miRNAs derived from algorithms using very similar search parameters are compared only about 50% overlap is seen [25]. One of our identified miRNAs, which is not reported by any other study, was recently identified experimentally ([26] present in miRbase version 10.0 and not 9.0) increasing confidence in our unique miRNAs. Notably, even though miRNAs from all species were used to search for human homologs, the candidate miRNAs discovered are homologs to genes in two species only, *M. musculus *and *R. norvegicus*, indicating better miRbase coverage for mouse and rat than for other species. Two examples of the non-registered human homolog miRNA genes are presented in Figure 2. miRbase mouse miR-764 sequence, which has no known registered homologs was used as input for miRNAminer search (with default parameters). The output reported a homolog (presumably hsa-miR-764; see Table 2), which is located in the second intron of Human serotonin receptor 2C (HTR2C; NM 000868; Figure 2A). The mouse miRNA homolog is located in an intron of the same gene (HTR2C; NM 008312) suggesting an evolutionary conserved co-expression of miRNA and its host gene [27-29]. Second in the list of non-registered human homolog miRNA genes (sorted according to BLAST e-values) is miR-763. This miRNA spans the longest complementary sequence out of the list, has the lowest RNA folding energy (Table 2) and shows high conservation between many species (Figure 2C). Interestingly, human miR-763 is harboured in an intron of the high mobility group AT-hook 2 oncogene (HMGA2; for review see [30,31]). Recent disrupted interplay between miRNAs and HMGA2 showed an increase in oncogenesis [32-34]. To regulate their targets, miRNAs bind to 'seed' regions in the 3'UTR, typically 6–7 nt long (nt 2 to nt 7 or 8 of the miRNA [15,35,36]; also see [37]). miR-763, possibly also co-expressed with its host gene [27-29], has a conserved binding site for its own harboured miRNA (nt 2 to 8 of the miRNA binds position 2192 in the 3'UTR which is conserved in human/mouse/rat). It is tempting to speculate a negative feedback regulatory role of newly identified human miR-763 and its oncogenic host when co-expressed in the same spatio-temporal context. To this end data from Expressed Sequence Tags (ESTs) supports this possibility (Figure 2C). Other identified miRNAs presented in Table 2 show high species conservation (for example, miR-670) or are located in exons (for example, miR-711) or exon-intron junctions (for example, miR-762). Interestingly, in a recent study involving deep sequencing, four of our human predicted miRNAs were confirmed (miR-760, 708, 543, and 665 [38], available in miRbase version 10.0). To conclusively confirm the presence of the identified candidates in the studied species, an experimental verification is required. However, the candidates identified by our method are close homologs to known miRNAs and as such are not required to meet as stringent criteria to be annotated as novel miRNAs [17]. In this study we looked at homolog genes which are genes related to each other by descent from a common ancestral DNA sequence. We do not segregate between orthologs, genes in different species that evolved from a common ancestral gene by speciation, and paralogs, genes separated by the event of genetic duplication. We cannot also rule out that similar miRNAs in different species have developed independently [39]. Our tool, which is based on evolutionary conservation, can only detect evolutionarily conserved miRNA genes. We are currently improving our algorithm to include multiple alignments of vertebrate miRNA sequences in order to better refine the boundaries of the miRNA precursor sequence.

For searches with relaxed parameters (reduced stringency) we suggest initially performing the following modifications: (i) do not 'Require seed conservation in mature miRNA (nt 2–8)' (uncheck box); (ii) increase 'maximal number of gaps in miRNA precursor alignment' from 10 (default) to 15; (iii) decrease 'minimal mature miRNA identity' from 0.8 (default) to 0.7; (iv) decrease 'minimal base pairing percentage in miRNA precursor' from 55 (default) to 40; and (v) change 'minimal/maximal length of precursor sequence (nt)' from 70/180 (default) to 50/250. In order to view miRNAs which are other than the top candidate we suggest increasing the 'number of results to report' from 1 (default) to 5. The parameters (i–v) above are listed in the order that would output an increasing total number of identified miRNAs. For example, reducing mature miRNA identity from 0.8 (default) to 0.7 increases miRNAs from 22 to 24 (9%) and 31 to 36 (16%) in human and mouse, respectively. On the other hand, we found that changing the length of the miRNA precursor from 70–180 nt (default) to 50–250 nt, added only 1 additional miRNA in human and none in mouse. This, however, might change when run in combination with other modified parameters. Altogether each of the modified parameters listed above will result, independently, in an average miRNA increase of 11% when tested on seven mammalian species.

We estimated miRNAminer's sensitivity (Table 3) and specificity. The sensitivity, on seven mammalian species, is 0.88. Sensitivity for a species is the portion of the species' miRNAs with known homologs that are detected by miRNAminer using miRNAs from all other species. We used only miRNAs which miRbase lists for more than one species. Sensitivity measures are higher in chimp (0.94), mouse (0.88) and rat (0.91) than in human (0.85). To estimate specificity, we used miRNAminer to search for miRNA homologs in *C. elegans*, which has a large evolutionary distance from the studied mammals. We treated as false positives all hits reported by miRNAminer that were not identified as homologs by previous studies. This conservative treatment may over-approximate the number of false positives. Using 1375 miRNAs from the seven studied mammalian species, miRNAminer detected, in *C. elegans*, two known homologs (let-7 and mir-124) and reported only five false positives.

**Table 3:** Sensitivity of miRNAminer.

Genome	Found	Not found	Sensitivity
Human	179	31	0.85
Chimpanzee	63	4	0.94
Mouse	184	24	0.88
Rat	154	15	0.91
Dog	5	0	1.00
Cow	58	14	0.81
Opossum	71	8	0.90

Total	714	96	0.88

The second (third) column presents the number of miRNAs with homologs that miRNAminer detects (does not detect) in each species.

## Conclusion

Several approaches to identify miRNA homologs have been previously described, both in plants [40], and in animals [5,41,42]. However, the only tool that is available as a web service, microHARVESTER [40], is targeted for plants. miRNAminer is the first available miRNA gene homolog search tool for animal genomes.

## Authors' contributions

NS conceived the study. SA, AK and NS planned and designed the algorithm and web-server. SA and AK wrote the code. NS analyzed the output. SA, AK and NS wrote the paper. All authors read and approved the manuscript.

## Availability and requirements

Project name: miRNAminer; Project home page: ; Operating system: Platform independent; Programming language: Java; License: Open source, see ; Code is available upon request. miRNAs identified using miRNAminer will be incorporated in next miRbase versions, see .

## Supplementary Material

Additional file 1 **Non-miRbase registered miRNAs**. A list of 790 miRNAs that were identified using miRNAminer. These miRNAs add more than 50% to the total count of miRNAs in the seven mammalian species tested: human, chimpanzee, mouse, rat, dog, cow and opossum, and are available at: Click here for file
